# Contributions to Operative and Mechanical Dentistry

**Published:** 1846-09

**Authors:** W. H. Elliot


### ARTICLE II.

*Contributions to Operative and Mechanical Dentistry.* By W. H. Elliot, D. D. S.

No. 9.

### ON THE WORKING OF STEEL, REFINING GOLD, AND MAKING SOLDERS.

Steel is the carbonate of iron; it contains about two per cent. of carbon.

Iron is made into steel by a process called cementation, which consists of burying bars of iron in a cement composed of charcoal, ashes and salt, so as to exclude the air, and then in keeping it heated to about 100 degrees Wedgwood for six or eight days, during which time, it absorbs a certain amount of carbon from the coal, and when removed from the furnace, it is found to be covered with blisters, and is therefore called *blister steel.* This steel is broken into small pieces, smelted, cast into bars, and then it receives the name of *cast steel.*

The fracture of *good cast steel* presents a fine uniform and silvery appearance, works easily and evenly, and tempers at a low heat.

Mechanics too often fall into the error of believing that the great secret of making good cutting instruments consists in tempering in some peculiar way; but experience has taught me, that if the steel has been once heated too hot or hammered too cold, any further attempts to produce a fine, firm edge will prove abortive.

*Cast steel* should never be heated above a bright red, or hammered after it ceases to be red by daylight. A want of attention to these rules, particularly in the construction of small instruments is sure to destroy the strength of the steel and render it useless for such purposes.

In flattening and curving small points, the anvil should be placed as near the fire as practicable, and during the process of hammering, the steel, instead of being allowed to touch the anvil should be held about an eighth of an inch above it, except at the instant it receives the blow from the hammer; in this way a small point will remain at the required temperature much longer than it would if it were allowed to rest upon the face of the anvil.

Steel should not be heated, for the purpose of hardening, above a cherry red, and the degree of hardness it acquires in quenching, depends principally upon the temperature of the bath into which it is plunged. I have used several kinds of hardening fluids, such as acidulated water, brine, oil, and quicksilver; but nothing can be relied upon with more certainty than pure cold water. The piece to be hardened should be heated evenly to a dark cherry color by the light of the sun, then plunged with some rapidity into the bath and allowed to remain until cold, it should then be polished, and the hardness of the temper reduced by heat.

Very few instruments are required as hard as the steel is found to be on removing it from the bath; besides, in this condition it breaks almost as easily as glass, but acquires, on drawing the temper, a remarkable toughness.

The several different tempers that are obtained by heating steel after it has once been hardened, are indicated correctly by the color the metal takes on, and these tempers are known by the names of the colors which indicate them; so that we say when speaking of a spring, it was drawn to a blue; or, of a lancet, it was drawn to a straw color; because these are the colors that indicate the proper temper for the spring and lancet respectively.

A piece of polished steel, when exposed to heat, takes on the following colors in succession, viz. 1st, a straw color—2d, yellow—3d, dark yellow—4th, copper color—5th, purple—6th, blue—7th, whitish blue. The first of these tempers is suitable for a gum lancet, the second for broaches and scaling instruments, the third for rose drills, the fourth for excavators, the sixth for springs, and the seventh for filling instruments; this last temper is used merely to give to the piece a greater degree of firmness.

In hardening an instrument, not only the cutting part, but as much of the shank as requires strengthening, should be plunged into the bath; and in drawing the temper the point should first be partly polished, so that the colors produed by heat, may readily be distinguished; and while the temper of the shank is being reduced to a whitish blue in the flame of a spirit lamp, the point should be held between the beaks of a pair of large pliers, to prevent it from being drawn too low, and then it may be released from the pliers and the heat allowed to run down until the point assumes the required shade.

Such points as do not admit of being polished after hardening, previously to being put into the fire, should be covered with a paste composed of a saturated solution of common salt, and some coarse flour. This coating, if carefully put on, prevents the point from oxydizing, or as mechanics term it, prevents the point from scaling. Rose drills, extracting screws, and all other denticulated instruments, are more or less injured by being heated to a redness without this covering.

The process of rendering gold malleable by melting and refining, is simple to those who witness it; yet the first attempts of the assayist are seldom perfectly successful, for the want of tact in the management of the fire, crucible, &c.; but the exercise of a little patience will overcome all difficulties. The directions that we shall give, are concise, yet sufficient for the purpose of the dental artist, and if followed out to the letter, will, in all cases, produce the desired result. The following implements are necessary for this purpose: a small draught furnace, a quantity of fine hard-wood coal, a clean crucible with a sheet-iron cover, a light crucible tongs, an ingot mould made of soap-stone, a little nitrate of potassa, carbonate of potassa, borax and oil. The fire-place of the furnace should be about ten inches in diameter, and eight or ten deep, this should be connected by means of a pipe with the chimney, so that a powerful draught may be made to pass through the coal. A blast furnace is objectionable for the reason that the bellows burns out the coal immediately under the crucible, and it is therefore constantly dropping down, which is not the case with the draught furnace; besides, the draught furnace produces a more even fire, a quality equally indispensable.

In preparing for a heat, the furnace should be filled about half full of coal, and after it is well ignited, it should be consolidated as much as practicable without choking the draught. The crucible containing the metal, and a little borax may then be set on, and more coal placed around and over it, the door of the furnace closed, and the damper opened; it should remain in this way until the gold is perfectly fused, the coal may then be removed from over the crucible, and a bit of nitrate of potassa dropped in, in quantity equal to the size of a pea to every ounce of gold, and the crucible immediately covered with a plate of iron; more gold may then be placed over and around the crucible, and the gold kept in a fused state at a high temperature, until the scoria ceases to pass off, which it will do in the course of five or six minutes. The ingot mould having been previously warmed, may be placed in a convenient position for pouring, and filled about half full of lamp oil. The iron cover may now be thrown off quickly, the crucible seized with the tongs, and at the same instant another small bit of nitrate of potassa should be thrown into it, and the gold rapidly, but carefully poured into the mould.

The ingot always cools first at the edges, and shrinks away from the middle; on that account the mould should be a little concave on the sides, so that the shrinking will not reduce the ingot thinner in the centre than at the edges.

Moulds of the best form will sometimes produce ingots of irregular thickness; such ingots should be brought to a uniform thickness under the hammer, using the common callipers as a guage; if this be neglected, the plate will be found imperfect at those points where the ingot was thinnest. The plate should be annealed occasionally during the process of hammering and rolling, and should be reduced about one number in thickness each time it passes between the rolls. If any lead, tin or zinc be mixed with the gold, the nitrate of potassa must be used in much larger quantities, and in that case, it is better to let the button cool in the bottom of the crucible; then break the crucible and melt it in a clean one for pouring, using borax and nitrate of potassa in very small quantities for the last melt.

In case the subject of assay be in the form of filings or dust, a magnet should be passed through it so as to remove every particle of iron, and then, instead of melting it with borax, it should be melted first with *carbonate of potassa*, and afterwards with nitrate of potassa in quantities proportioned to the necessities of the case as before directed; carbonate of potassa is the only flux that will bring all the small particles of metal into one mass; without it, a great portion of the gold will be found among the scoria, adhering to the sides of the crucible in the form of small globules. This process of refining, answers equally as well for silver as gold.

Lead, tin or zinc, alloyed with gold, destroys its malleability, and it is for the purpose of freeing these metals principally, that the nitrate of potassa is used. If the alloy contain copper, a portion of that metal will also be carried away in the scoria, but silver remains untouched. The silver and remaining portion of copper, however, may be *parted* from the gold by another very simple process, which consists of dissolving these metals out of the alloy by nitric acid. To do this successfully, there are several rules to be observed, viz. first, the proportion of silver of the alloy, should be three times the weight of the gold. Second, the copper should not exceed one-third the weight of the gold, and lastly, the strength of the acid should be such as to give it a specific gravity, of about 1.310; or in other words, a pint of acid should weigh at least twenty-one ounces. If the alloy contains less than twice as much silver as gold, the particles of gold will protect the silver and copper from the action of the acid, and if it contains too much copper, the parting will be found much more difficult.

My own experience teaches me, that it is better to separate all or nearly all of the copper from the alloy, by melting with nitrate of potassa, before any attempt be made to part the silver and gold by acid.

For separating, the alloy should be rolled into thin sheets and placed in a deep glass or earthen vessel, and two or three times its weight of nitric acid poured upon it; the whole may then be heated until ebulition takes place, and after boiling five minutes the acid should be carefully poured off, and new acid used for the same length of time; then the last acid may be poured with the first, and the gold washed with pure water. The silver of the alloy will now be found in the acid, and may be thrown down in its pure state, by plunging it into the solution plates of copper.

The silver powder may be collected, washed, and melted with a little carbonate of potassa, the gold may also be melted with the same flux, and both metals will be found to be in a perfectly pure state; the copper, if any there were in the alloy, is still held in the solution, and may be deposited in its turn by plates of zinc.

There is another beautiful method of refining gold by cupellation; but this requires close attention, and considerable experience, and on that account, is not much used for small assays.

The metals used in making solders, should be refined separately before weighing, and then melted in a clean crucible with a little borax, and as soon as the metals are well fused, the crucible should be slightly shaken, and the alloy immediately poured into the mould. The ingot of solder should be hammered, rolled, and occasionally annealed with as much care as if it were a plate of gold.

Any attempt to refine solder, would of course take away a portion of its copper, and consequently, destroy its proportion ; on this account, it is better to use pure gold, pure silver, and copper selected from the Russian coin.

If, by accident, a quantity of solder has become impure, so as to be unfit for use, it should be weighed correctly and then melted with nitrate of potassa, and allowed to cool in the bottom of the crucible, the crucible may then be broken and the button weighed the second time, and as much fine copper added as the solder is found to have lost by refining.

Zinc should never be used in making gold solders, for the reason that the better qualities of gold will melt at a comparatively low heat when brought in contact with solder containing zinc, thereby subjecting the artist to the most annoying accidents.

Potter's receipt for making solder, so often sold as a secret to dentists in the country, gives seventy-two parts gold, five of zinc, and four of copper. This solder does not flow well, is liable to accidents when used on fine gold, and is much more expensive than solders that offer equal resistance to oxygen which are composed of gold, silver and copper.

I have made and used several kinds of solder, and of course, have my favorite receipts, but I know of none that possesses the requisite qualities in a more eminent degree than those made from the receipt of Professor Harris *

### ARTICLE III.

*Dissertation on Preparation of a Cavity in a Tooth, preparatory to Plugging.* By W. H. DWINELLE. Read before the American Society of Dental Surgeons, at its Seventh Annual Meeting, held in the City of New York, August 4th, 1846.

*Mr, President and Gentlemen :*

I AM aware that I am about to propose a course of practice to you, wholly at variance with the present system, and which, at

* Principles and Practice of Dental Surgery, page 579.

